# Clinical predictors of radiation-induced lymphopenia in patients receiving chemoradiation for glioblastoma: clinical usefulness of intensity-modulated radiotherapy in the immuno-oncology era

**DOI:** 10.1186/s13014-019-1256-6

**Published:** 2019-03-27

**Authors:** Hwa Kyung Byun, Nalee Kim, Hong In Yoon, Seok-Gu Kang, Se Hoon Kim, Jaeho Cho, Jong Geol Baek, Jong Hee Chang, Chang-Ok Suh

**Affiliations:** 1 Department of Radiation Oncology, Yonsei Cancer Center, Yonsei University College of Medicine, 50-1 Yonsei-ro, Seodaemun-gu, Seoul, 03722 Republic of Korea; 2 Department of Neurosurgery, Yonsei University College of Medicine, Seoul, Republic of Korea; 3 Department of Pathology, Yonsei University College of Medicine, Seoul, Republic of Korea; 4 Department of Radiation Oncology, CHA Bundang Medical Center, CHA University, Seongnam, Republic of Korea

**Keywords:** Glioblastoma, Lymphopenia, Immunotherapy, Radiation, Chemotherapy, Treatment-related toxicity

## Abstract

**Background:**

Immunotherapy is currently being examined as a treatment modality for glioblastoma. Maintaining an optimal total lymphocyte count (TLC) after radiotherapy (RT) and using temozolomide may be beneficial in optimizing immunotherapy. However, conventional temozolomide-based chemoradiation is known to induce immunosuppressive effects, including lymphopenia. Therefore, this study aimed to identify potential clinical predictors of acute severe lymphopenia (ASL) in patients receiving chemoradiation for glioblastoma.

**Methods:**

We identified patients with glioblastoma treated with RT plus temozolomide from 2006 to 2017. ASL was defined as a TLC of < 500/μL within 3 months after initiating RT. Independent predictors of ASL were determined using logistic regression.

**Results:**

A total of 336 patients were evaluated. Three-dimensional conformal RT (3D-CRT) and intensity-modulated RT (IMRT) were used in 186 (55.4%) and 150 patients (44.6%), respectively. TLC decreased during RT and remained persistently low during the 1-year follow-up, whereas the levels of other blood cell types recovered. In total, 118 patients (35.1%) developed ASL. During a median follow-up of 19.3 months, patients with ASL showed significantly worse overall survival than did those without ASL (median, 18.2 vs. 22.0 months; *P* = .028). Multivariable analysis revealed that increased planning target volume (PTV) was independently associated with increased ASL incidence (hazard ratio [HR], 1.02; 95% confidence interval [CI], 1.00–1.03; *P* = .042), while IMRT was independently associated with decreased ASL incidence (HR, 0.48; 95% CI, 0.27–0.87; *P* = .015). A propensity-matched comparison showed that the incidence of ASL was lower with IMRT than with 3D-CRT (20% vs. 37%; *P* = .005).

**Conclusions:**

IMRT and low PTV were significantly associated with decreased ASL incidence after RT plus temozolomide for glioblastoma. An IMRT-based strategy is necessary to enhance treatment outcomes in the immune-oncology era.

**Electronic supplementary material:**

The online version of this article (10.1186/s13014-019-1256-6) contains supplementary material, which is available to authorized users.

## Background

Radiotherapy (RT)-induced lymphopenia (i.e., a reduction in the total lymphocyte count [TLC]) has been reported in various types of tumors, such as glioblastomas, pancreatic cancer, and lung cancer [1–7]. Although radiation has local effects, RT to peripheral organs can result in irradiation of a substantial proportion of circulating lymphocytes during multifraction treatments [8]. Recently, several studies have demonstrated that partial brain RT can contribute to systemic lymphopenia [5–8]. RT-induced lymphopenia is associated with poor survival in patients with high-grade gliomas who underwent standard therapy with RT and temozolomide [5, 6].

Despite multimodal treatment involving surgery, RT, and temozolomide, glioblastoma has a poor prognosis and almost all patients with glioblastoma eventually experience disease relapse [9]. Although repeat surgery, re-irradiation, and pharmacological treatment have been performed in the recurrent setting, evidence that any therapeutic intervention has a major effect on survival is lacking [10, 11]. Accordingly, different immunotherapy modalities for glioblastoma are being actively investigated, spurred on by advances in immuno-oncology for other tumor types [12]. However, conventional temozolomide-based chemoradiation has immunosuppressive effects, including lymphopenia [5, 6, 13]. As lymphocytes are important mediators of the immune response to cancer, such iatrogenic immunosuppression can limit the administration of immunotherapy.

In this context, identifying and modifying the factors associated with RT-induced lymphopenia can help maintain an optimal TLC, which may facilitate a synergistic effect between RT and immunotherapy. Accordingly, maintaining an optimal TLC may effectively improve treatment outcomes in patients with glioblastoma. Therefore, this study aimed to examine the potential clinical predictors of treatment-related lymphopenia in patients with glioblastoma treated with RT plus temozolomide.

## Methods

### Patients

Between February 2006 and January 2017, we identified consecutive patients with histologically confirmed glioblastoma treated with temozolomide-based chemoradiation. We excluded patients who received whole-brain RT for gliomatosis cerebri or extensive disease, those who received whole-ventricle RT with a suspicion of ventricular seeding, and those without peripheral blood cell count results before and within 3 months after initiating RT.

### Treatments

Patients underwent either tumor resection or stereotactic biopsy. Thereafter, all patients received concurrent chemoradiation with temozolomide (75 mg/m^2^ of body surface area per day, 7 days per week from the first to the last day of RT), followed by six cycles of adjuvant temozolomide (150–200 mg/m^2^ for 5 days during each 28-day cycle).

Regarding the target volume, a limited field or a standard field was used based on the physician’s preference [14]. The gross tumor volumes (GTVs) in both fields comprised the resection cavity and any residual contrast-enhancing tumor on immediate postoperative magnetic resonance imaging (MRI) scans obtained within 48 h after surgery. When delineating the GTV, we added a 0.5–1-cm margin to compensate for irregularity and uncertainty. The clinical target volume (CTV) in the standard field included peritumoral edema, which was detected on T2-weighted fluid-attenuated inversion recovery postoperative MRI scans (peritumoral edema + 1–1.5 cm margin). The CTV in the limited field was delineated by adding a 1.5-cm margin to the GTV, regardless of the presence of peritumoral edema. In the three-dimensional conformal RT (3D-CRT) plan, a 3-mm margin—for setup uncertainty—was applied to create the planning target volume (PTV). PTV1 and PTV2 were defined as the CTV plus a 3-mm margin and the GTV plus a 3-mm margin, respectively. In 3D-CRT, 46 Gy in 23 fractions to the PTV1 and a sequential boost of 14 Gy in 7 fractions to the PTV2 were prescribed. No PTV margin was added in the intensity-modulated RT (IMRT) plan, and the PTV1 and PTV2 were the same as the CTV and GTV, respectively. Instead, when IMRT was administered, megavoltage or kilovoltage cone-beam computed tomography image guidance was performed before each treatment session for all patients. In IMRT, 51 Gy in 30 fractions to the PTV1 and 60 Gy in 30 fractions to the PTV2 were prescribed using a simultaneous integrated boost technique.

### Assessment of lymphopenia

Peripheral blood counts were typically assessed every week during RT and then every 1–3 months after RT for 1 year. Lymphopenia was graded using the Common Terminology Criteria for Adverse Events criteria, version 4.03. A TLC of the lower limit of the normal value to 800/μL was categorized as grade I, 800–500/μL as grade 2, 500–200/μL as grade 3, and < 200/μL as grade 4. Acute severe lymphopenia (ASL) was defined as a TLC of < 500/μL (grade 3/4 toxicity) within 3 months after beginning RT, as previously described [7].

### Assessment of other baseline characteristics

Subventricular zone involvement was assessed via preoperative MRI using a standardized spatial classification system [15]. The extent of resection was evaluated using immediate postoperative gadolinium-enhanced T1-weighted MRI scans and was categorized as total (absence of any visible contrast-enhanced portions), subtotal (≥90% of the tumor removed), partial (< 90% of the tumor removed), or biopsy. The DNA methylation status of the CpG islands on the MGMT promoter and the *IDH1-R132H* mutation (*IDH1* mutation) status were also examined.

### Statistical analysis

Overall survival (OS) rates were calculated from the start date of treatment to the date of death or the latest follow-up visit by using the Kaplan-Meier method and compared using the log-rank test. Univariate and multivariate analyses using Cox and logistic regression were performed to identify the predictors of OS and the development of ASL, respectively. Factors with a *P*-value of <.05 in the univariate analyses were included in the subsequent multivariate analysis. All tests were two-sided, and significance was set at *P* < .05. The changes in blood cell counts over time were plotted using the R package “hexbin” [16]. Propensity score matching between the 3D-CRT and IMRT groups was performed by using 1:1 nearest neighbor (greedy-type) analysis with a caliper width of 0.2 standard deviations of the logit distance measure by using the R package “MatchIt” [17]. Matching covariates were selected based on their potential impact on the development of ASL according to logistic regression analysis. These covariates included sex, extent of resection, *IDH1* mutation, PTV1, baseline TLC, and the cumulative dose of temozolomide. All statistical analyses were performed using the Statistical Package for Social Sciences version 23.0 (IBM SPSS Statistics, Armonk, NY) and R software version 3.4.1 (R Foundation for Statistical Computing, Vienna, Austria; https://www.R-project.org/).

## Results

### Patients and treatments

Of 374 consecutive patients, we excluded 38 because they received whole-brain RT for gliomatosis cerebri or extensive disease (*n* = 20), received whole-ventricle RT with a suspicion of ventricular seeding (*n* = 15), and had no peripheral blood cell count results before and within 3 months after initiating RT (*n* = 3). Thus, 336 patients who received partial brain RT were selected for the final analysis. Tumor resection was performed in 314 patients (93.5%), while stereotactic biopsy was performed in 22 (6.5%). Most patients (*n* = 331, 98.5%) received a conventionally fractionated regimen with a total dose of 60 Gy in 30 fractions. Regarding the target volume, a limited field and a standard field were used in 49 (14.6%) and 287 patients (85.4%), respectively. A total of 186 (55.4%) and 150 (44.6%) patients received 3D-CRT and IMRT, respectively. Significant inter-group differences were observed between the 3D-CRT and IMRT groups in the age, sex, extent of resection, *IDH1* mutation status, PTV, and number of fractions (all *P* < .05). The median PTV1 for the 3D-CRT and IMRT groups was 422 cm^3^ (range, 74–1080 cm^3^) and 375 cm^3^ (range, 71–1041 cm^3^), respectively (*P* < .001). The mean cumulative dose of temozolomide for 3 months since the start of chemoradiation was not significantly different between the groups (3497 mg/m^2^ vs. 3684 mg/m^2^; *P* = .117). The baseline patient characteristics and treatment details are provided in Table 1.

**Table 1:** Patient and treatment characteristics

	Total (*n* = 336)	3D-CRT (*n* = 186)	IMRT (*n* = 150)	P*
Age, years, median (range)	58 (16–79)	59 (16–79)	56 (16–79)	0.004
Sex, n (%)				
Female	149 (44.3)	96 (51.6)	53 (35.3)	0.003
Male	187 (55.7)	90 (48.4)	97 (64.7)	
KPS, n (%)				
≤ 70	172 (51.2)	104 (55.9)	68 (45.3)	0.156
80	82 (24.4)	41 (22)	41 (27.3)	
≥ 90	82 (24.4)	41 (22)	41 (27.3)	
Extent of resection, n (%)				
Total	206 (61.3)	109 (58.6)	97 (64.7)	0.009
Subtotal/Partial	108 (32.1)	70 (37.6)	38 (25.3)	
Biopsy	22 (6.5)	7 (3.8)	15 (10)	
*IDH1* mutation, n (%)				
No	250 (74.4)	117 (62.9)	133 (88.7)	< 0.001
Yes	18 (5.4)	6 (3.2)	12 (8)	
unknown	68 (20.2)	63 (33.9)	5 (3.3)	
*MGMT,* n *(%)*				
Unmethylated	212 (63.1)	115 (61.8)	97 (64.7)	0.592
Methylated	124 (36.9)	71 (38.2)	53 (35.3)	
Subventriclular zone, n (%)				
Uninvolved	206 (61.3)	122 (65.6)	84 (56)	0.073
Involved	130 (38.7)	64 (34.4)	66 (44)	
PTV1 volume, cm^3^, median (range)	403 (71–1080)	422 (74–1080)	375 (71–1041)	<.001
PTV2 volume, cm^3^, median (range)	113 (6–475)	119 (12–475)	103 (6–468)	0.002
Total dose, Gy, median (range)	60 (41–72.5)	60 (54–70)	60 (41–72.5)	0.073
No. of fractionation, median (range)	30 (15–35)	30 (23–35)	30 (15–33)	<.001
Baseline TLC, /μL, median (range)	1370 (300–3740)	1365 (300–3740)	1385 (410–3640)	0.648
Baseline TLC, /μL, n (%)				
< 1000	81 (24.1)	45 (24.2)	36 (24)	0.893
≥ 1000	255 (75.9)	141 (75.8)	114 (76)	
The cumulative dose of temozolomide^a^, mg/m^2^, median (range)	3588 (722–6267)	3497 (856–6267)	3684 (722–5599)	0.117

*Abbreviations: 3D-CRT* three-dimensional conformal radiotherapy, *IMRT* intensity-modulated radiotherapy, *KPS* Karnofsky performance status, *PTV* planning target volume, *TLC* total lymphocyte count

*P-values were calculated for the comparison of the 3D-CRT and IMRT groups

^a^The cumulative doses of temozolomide for 3 months from the start of chemoradiation were calculated

### Blood cell counts

**Fig. 1:**
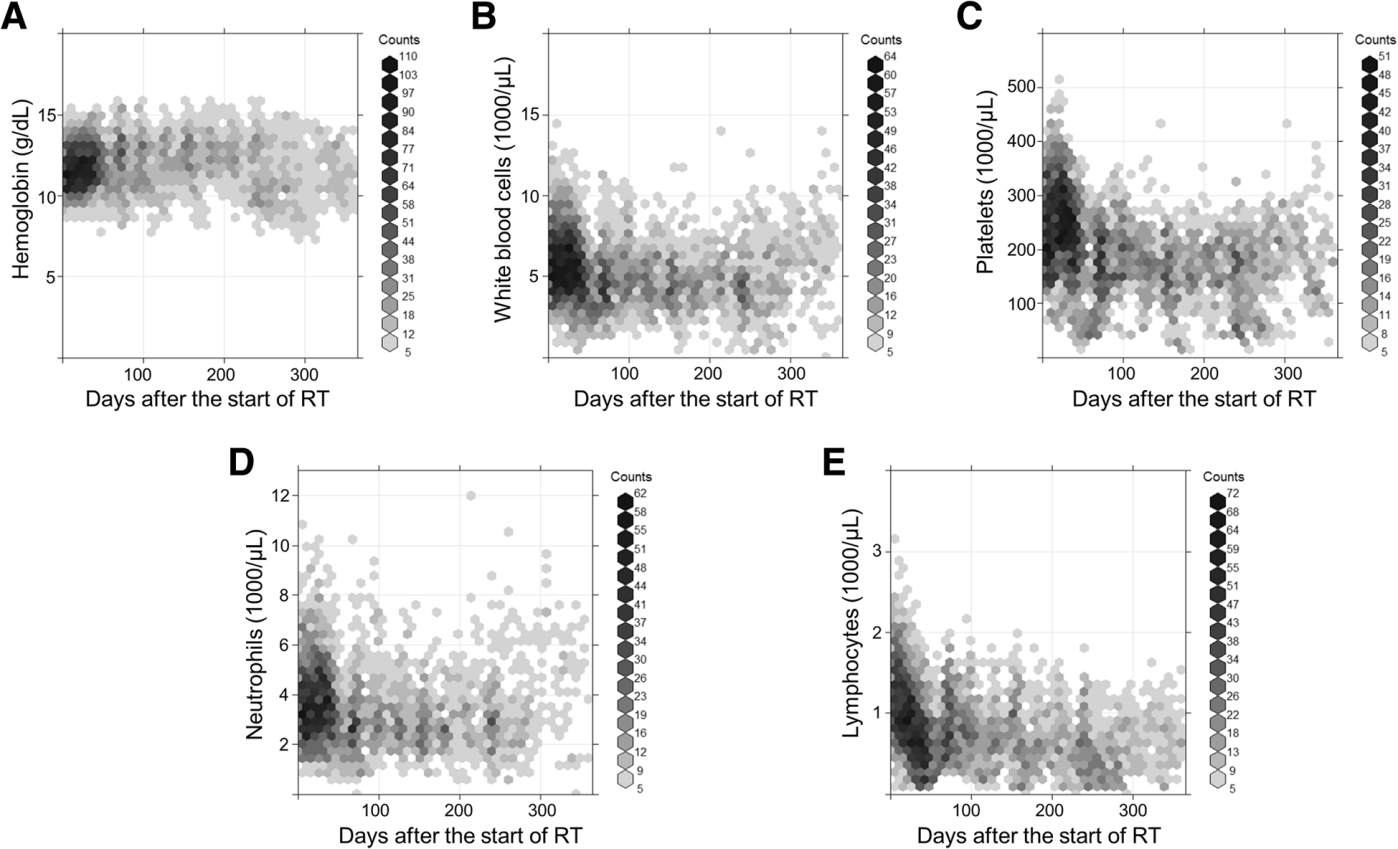
Scatter plot indicating changes in blood cell counts. Hemoglobin concentration (**a**), and white blood cell (**b**), platelet (**c**), neutrophil (**d**), and lymphocyte counts (**e**) over the 1-year follow-up. Density was computed using the R package “hexbin,” with *N* = 35 bins partitioning the data range. Compared with the count of the other blood cell types, the total lymphocyte count was markedly decreased after the start of radiotherapy (RT) and remained low during the 1-year follow-up

During the 1-year follow-up after the initiation of RT, the median number of blood tests performed per patient was 30 (range, 7–217). Changes in hemoglobin concentrations and blood cell counts, including those of white blood cells, platelets, neutrophils, and lymphocytes, are shown in Fig. 1. The TLC decreased during chemoradiation and remained persistently low over the follow-up period. In contrast, hemoglobin concentrations did not decrease significantly after chemoradiation. The counts of white blood cells, platelets, and neutrophils decreased during chemoradiation but showed a tendency to improve during a year of follow-up.

In total, 118 patients (35.1%) developed ASL. The median TLC at baseline was 1370/μL (range, 300–3740/μL), with 3.0% of patients having grade ≥ 3 lymphopenia. At 1, 3, and 12 months after the initiation of RT, the median TLC was 1020/μL (range, 110–3230/μL), 1120/μL (range, 170–3070/μL), and 980/μL (range, 30–2680/μL), respectively, and 10.1, 5.5, and 10.3% of patients developed grade ≥ 3 lymphopenia.

### Survival analysis

**Fig. 2:**
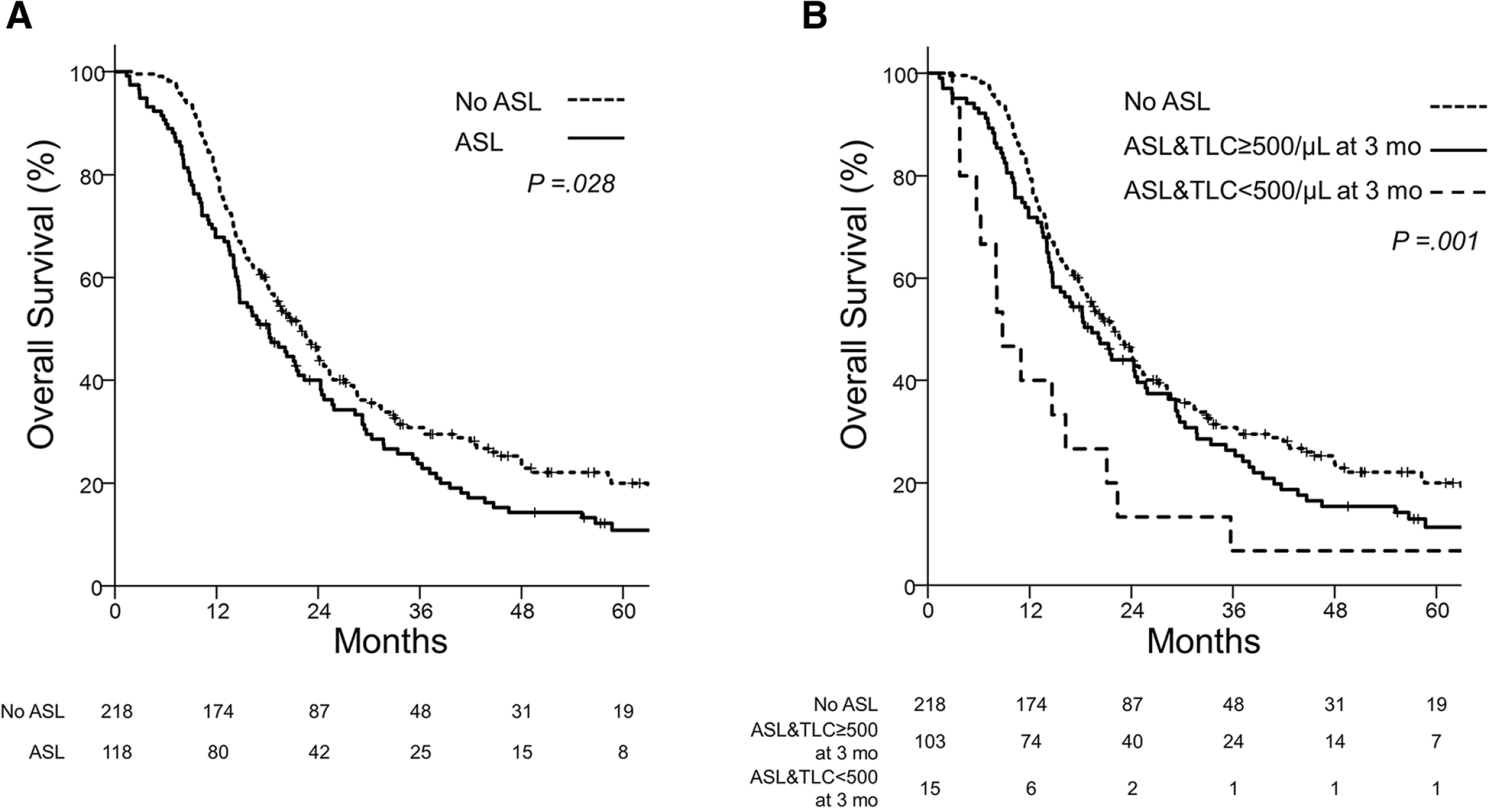
Kaplan-Meier estimates of overall survival according to ASL (**a**), and ASL and TLC (**b**). ASL, acute severe lymphopenia; TLC, total lymphocyte count

During a median follow-up of 19.3 months (range, 1.4–145.0 months), the median OS was 20.5 months. Patients with ASL had significantly poorer OS than did those without ASL (median, 18.2 vs. 22.0 months; *P* = .028; Fig. 2a). On Cox regression multivariate analysis, lower age, total resection, *IDH1* mutation, MGMT methylation, and absence of subventricular zone involvement significantly indicated favorable OS (all *P* < .05). ASL was significantly associated with poorer OS on univariate analysis (*P* = .028), but it lost its significance for OS after adjustment for other confounders (*P* = .756; Table 2). When Cox regression analyses were separately conducted for the 3D-CRT and IMRT groups, ASL was not an independent prognostic factor for OS in both groups (Additional file 1: Tables S1 and S2).

**Table 2:** Univariate and multivariate Cox regression analyses for overall survival

	Univariate HR (95% CI)	*P*	Multivariate HR (95% CI)	*P*
Patient-related factor				
Age (per 1 year)	1.01 (1.00–1.02)	0.005	1.02 (1.01–1.03)	0.003
Sex (Male vs. Female)	1.02 (0.80–1.30)	0.889		
KPS				
(80 vs. ≤70)	1.06 (0.79–1.42)	0.697		
(≥90 vs. ≤70)	0.78 (0.58–1.06)	0.112		
Baseline TLC (< 1000/μL vs. ≥1000/μL)	1.25 (0.94–1.64)	0.119		
Tumor-related factor				
Extent of resection				
(Subtotal/Partial vs. Total)	1.78 (1.38–2.31)	<.001	1.75 (1.33–2.31)	<.001
(Biopsy vs. Total)	1.69 (1.06–2.70)	0.028	1.50 (0.93–2.43)	0.095
*IDH1* mutation				
(Yes vs. No)	0.25 (0.11–0.55)	<.001	0.30 (0.13–0.69)	0.005
(Unknown vs. No)	1.14 (0.86–1.52)	0.360	0.97 (0.71–1.31)	0.818
MGMT (Methylated vs. Unmethylated)	0.46 (0.35–0.60)	<.001	0.48 (0.37–0.63)	<.001
Subventriclular zone (Involved vs. Uninvolved)	1.57 (1.23–2.01)	<.001	1.52 (1.17–1.96)	0.001
Treatment-related factor				
The cumulative dose of temozolomide (per 100 mg/m^2^)	1.00 (0.99–1.00)	0.260	1.00 (0.99–1.01)	0.597
PTV1 volume (per 10 cm^3^)	1.01 (1.00–1.01)	0.132		
PTV2 volume (per 10 cm^3^)	1.02 (1.00–1.03)	0.029	1.01 (0.99–1.02)	0.305
Radiotherapy modality (IMRT vs. 3D-CRT)	0.91 (0.71–1.17)	0.483		
Total dose (per 1 Gy)	0.98 (0.95–1.01)	0.187		
No. of fractionation (per 1)	0.99 (0.94–1.05)	0.845		
Acute severe lymphopenia (Yes vs. No)	1.32 (1.03–1.69)	0.028	1.04 (0.81–1.35)	0.756

*Abbreviations: 3D-CRT* three-dimensional conformal radiotherapy, *CI* confidence interval, *HR* hazard ratio, *IMRT* intensity-modulated radiotherapy, *KPS* Karnofsky performance status, *PTV* planning target volume, *TLC* total lymphocyte count

When a subgroup of 15 patients who showed grade ≥ 3 lymphopenia at the 3-month time point was included in the multivariate analysis, having grade ≥ 3 lymphopenia at the 3-month time point was associated with poor OS after adjustment for other confounders (hazard ratio [HR], 2.44; 95% confidence interval [CI], 1.42–4.19; *P* = .001; Additional file 1: Table S3). This subgroup also showed worse OS than did patients who had no ASL or had recovered from ASL at the 3-month time point (median OS, 8.8 vs. 19.3 vs. 22.0 months; *P* = .001; Fig. 2b).

### Predictors of ASL

The results of univariate and multivariate logistic regression analyses for ASL are shown in Table 3. Among patient- and tumor-related factors, female sex (*P* < .001) and subtotal or partial resection (*P* = .001) were independently associated with increased ASL incidence. Among the treatment-related factors, an increased PTV1 (per 10 cm^3^; HR, 1.02; 95% CI, 1.00–1.03; *P* = .042) was independently associated with increased ASL incidence. In contrast, the use of IMRT (HR, 0.48; 95% CI, 0.27–0.87; *P* = .015) was independently associated with decreased ASL incidence.

**Table 3:** Univariate and multivariate logistic regression analyses for acute severe lymphopenia

	Univariate HR (95% CI)	*P*	Multivariate HR (95% CI)	*P*
Patient-related factor				
Age (per 1 year)	1.02 (1.00–1.03)	0.098		
Sex (Male vs. Female)	0.30 (0.18–0.47)	<.001	0.33 (0.19–0.55)	<.001
KPS				
(80 vs. ≤70)	0.90 (0.53–1.56)	0.716		
(≥90 vs. ≤70)	0.54 (0.30–0.97)	0.038		
Baseline TLC (< 1000/μL vs. ≥1000/μL)	1.83 (1.09–3.07)	0.021	1.69 (0.94–3.03)	0.082
Tumor-related factor				
Extent of resection				
(Subtotal/Partial vs. Total)	2.85 (1.75–4.64)	<.001	2.40 (1.40–4.11)	0.001
(Biopsy vs. Total)	1.57 (0.62–3.94)	0.338	2.43 (0.84–6.98)	0.100
*IDH1* mutation				
(Yes vs. No)	0.64 (0.21–2.01)	0.447	0.75 (0.22–2.57)	0.649
(Unknown vs. No)	2.68 (1.56–4.64)	<.001	1.83 (0.93–3.62)	0.079
MGMT (Methylated vs. Unmethylated)	0.69 (0.43–1.11)	0.122		
Subventriclular zone (Involved vs. Uninvolved)	1.27 (0.80–2.00)	0.308		
*Treatment-related factor*				
The cumulative dose of temozolomide (per 100 mg/m^2^)	0.98 (0.97–1.00)	0.087	0.99 (0.97–1.02)	0.530
PTV1 volume (per 10 cm^3^)	1.02 (1.00–1.03)	0.012	1.02 (1.00–1.03)	0.042
PTV2 volume (per 10 cm^3^)	1.02 (1.00–1.05)	0.083		
Radiotherapy modality (IMRT vs. 3D-CRT)	0.32 (0.19–0.51)	<.001	0.48 (0.27–0.87)	0.015
Total dose (per 1 Gy)	0.95 (0.89–1.01)	0.072		
No. of fractionation (per 1)	0.99 (0.90–1.08)	0.749		

*Abbreviations: 3D-CRT* three-dimensional conformal radiotherapy, *CI* confidence interval, *HR* hazard ratio, *IMRT* intensity-modulated radiotherapy, *KPS* Karnofsky performance status, *PTV* planning target volume, *TLC* total lymphocyte count

**Fig. 3:**
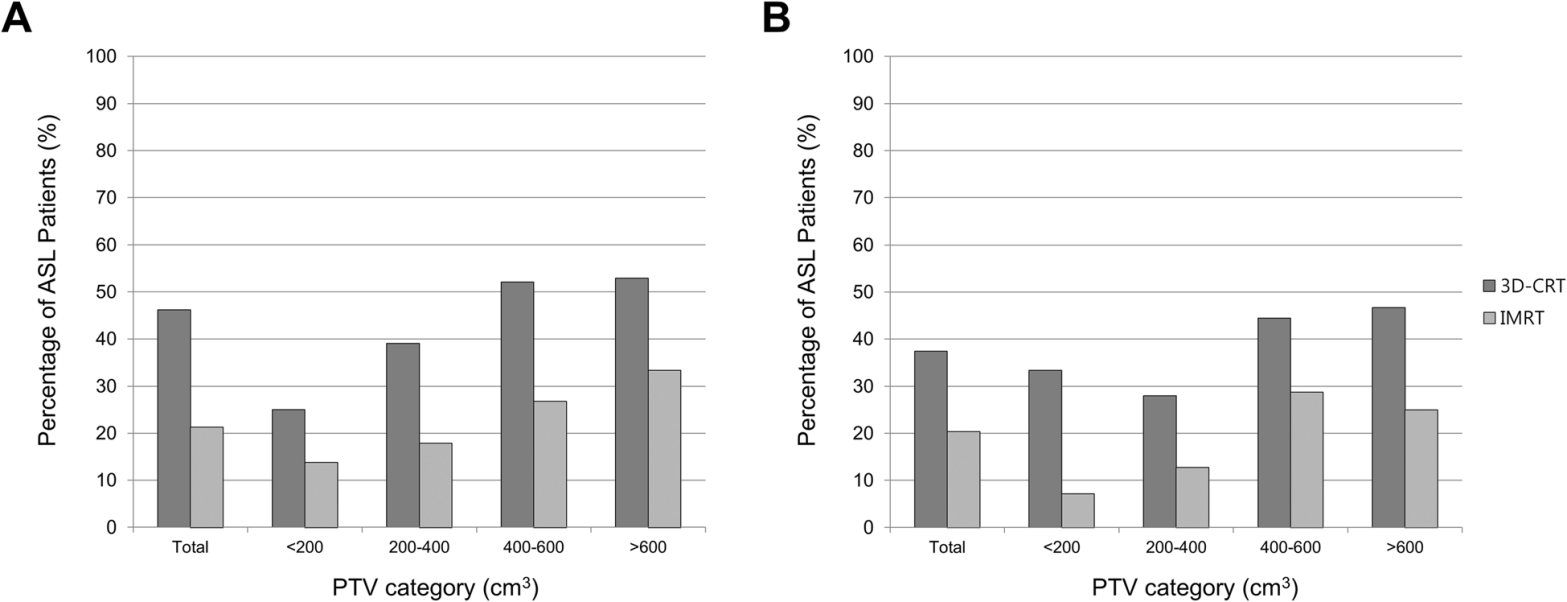
Incidence rates of ASL according to the PTV and radiotherapy modality. All patients (**a**); Propensity-matched patients (**b**). ASL, acute severe lymphopenia; 3D-CRT, three-dimensional conformal radiotherapy; IMRT, intensity-modulated radiotherapy; PTV, planning target volume

When patients were stratified according to the PTV1, the incidence of ASL increased as the PTV1 increased. In addition, in each PTV1 subgroup, patients treated with IMRT had a lower ASL incidence. With a PTV1 of < 200, 200–400, 400–600, and > 600 cm^3^, ASL developed in 14, 18, 27, and 33% of patients in the IMRT group, respectively, and in 25, 39, 52, and 53% of patients in the 3D-CRT group, respectively. In total, ASL developed in 21.3 and 46.2% of the patients in the IMRT and 3D-CRT groups, respectively (*P* < .001; Fig. 3a).

Propensity score matching was performed to adjust for significant differences in the baseline characteristics between the IMRT and 3D-CRT groups. After matching, sex, extent of resection, *IDH1* mutation, PTV1, baseline TLC, and the cumulative dose of temozolomide were well balanced between the groups (Additional file 1: Table S4). Among the 113 matched pairs, ASL developed in 23 (20.4%) and 42 (37.2%) patients in the IMRT and 3D-CRT groups, respectively (*P* = .005). In each PTV1 subgroup among the propensity-matched cohort, patients treated with IMRT had a lower ASL incidence (Fig. 3b).

## Discussion

In this study, we confirmed previous evidence regarding treatment-induced lymphopenia by using a relatively large population of patients with glioblastoma who received standard RT and chemotherapy [5–7]. The present study showed that cell counts of various blood cell types change over time after chemoradiation. Among them, the decrease in the lymphocyte count after chemoradiation persisted for a year, while other blood cell types, such as white blood cells, platelets, and neutrophils, showed recovery patterns and the count of red blood cells did not decrease after chemoradiation. Grossman et al. [5] showed that the CD4 count had the lowest value at 2 months after RT and remained persistently low after temozolomide-based chemoradiation in 96 patients with high-grade glioma. Moreover, this chronicity of lymphopenia was consistent with the lymphopenia pattern in other types of cancers such as pancreatic cancer and lung cancer [1]. Our findings confirmed such a pattern of treatment-related lymphopenia in 323 patients with glioblastoma and showed that this pattern was unique to changes in the lymphocyte count.

The clinical significance of treatment-related lymphopenia in patients with glioblastoma has drawn more attention in the modern immuno-oncology era. The therapeutic options for patients with recurrent glioblastoma are currently limited, but repeat surgery, RT, and pharmacological treatment with alkylating agents or bevacizumab have been performed [10, 11]. Nevertheless, a substantial proportion of patients do not receive any second-line anticancer therapy [18, 19]. Accordingly, different types of immunotherapy are being actively examined as novel approaches for the treatment of recurrent glioblastoma, including the glioblastoma vaccine, oncolytic viral therapy, chimeric antigen receptor T-cell therapy, and immune-checkpoint inhibitors [12]. For these immunotherapies, preserving the optimal lymphocyte count is essential, as lymphocytes play a fundamental role in cell-mediated immunologic destruction of cancers [20]. However, conventional first-line therapies for glioblastoma, including RT and temozolomide, have immunosuppressive effects. Moreover, as shown in the TLC patterns after chemoradiation in our study, the immunosuppressive effects can become chronic, i.e., the effects can even limit the applicability of immunotherapy as a second-line treatment option after relapse that mostly occurs after a few months or years. Therefore, efforts to identify the risk factors for ASL are important for establishing optimal chemoradiation strategies to preserve the TLC, which may have a potential synergistic effect with immunotherapy as a second-line treatment.

On examining various clinical and therapeutic factors, we found that female sex, subtotal/partial resection, increased PTV, and the use of 3D-CRT were independently associated with the development of ASL. Interestingly, the association between female sex and ASL has also been shown in previous studies on temozolomide-based chemoradiation for high-grade glioma [6, 7]. Although this could be attributed to differences in the pharmacokinetics and pharmacodynamics between sexes, the exact mechanism remains unknown [21].

A mathematical computation model has supported the association between local RT for body parts without the bone marrow or lymphatic tissue and systemic lymphopenia [8]. This model demonstrated that the mean dose to circulating lymphocytes is approximately 2 Gy and that nearly all the circulating blood receives at least 0.5 Gy during a typical course of RT for glioblastoma. Considering that lymphocytes are the most radiosensitive cells among all blood cell types and that the LD50 (lethal dose required to reduce the surviving fraction of lymphocytes by 50%) is only 2 Gy, this effect of RT on the circulating blood volume can cause ASL [22]. This model also examined two different PTVs (4.2 and 268 cm^3^) for glioblastoma and showed a large difference in the percent of blood receiving at least 0.5 Gy according to the target volume size. Our study confirmed the finding of this theoretical model regarding the effect of conventionally fractionated RT and PTV size on treatment-related lymphopenia by using clinical data of patients with glioblastoma.

We demonstrated that IMRT significantly reduced the development of ASL in patients receiving a conventionally fractionated regimen for glioblastoma. Compared to 3D-CRT, IMRT improves the dose distribution to the target volume as well as reduces the dose to normal tissues, such as normal brain tissues, optic structures, and the brain stem [23]. We collected data on the low-dose distribution to the brain and found that although V_0.5 Gy_ and V_3 Gy_ were not significantly different between the 3D-CRT and IMRT groups, V_5 Gy_, V_10 Gy_, and V_25 Gy_ were significantly lower in the IMRT groups (Additional file 1: Table S5). However, the irradiation volume of blood can become high in IMRT because IMRT usually takes a longer time than 3D-CRT. Accordingly, a precise mathematical method is needed to calculate the accurate dose for individual cases, which should be evaluated in further studies. IMRT has been adopted for the routine clinical treatment of many types of cancers including brain tumors [24]. However, compared to the use of IMRT for head and neck cancer [25], the clinical significance of IMRT for glioblastoma has not been well established to date. In this study, we showed the benefit of using IMRT for treating brain tumor in terms of preserving immunity. As immunotherapy is rapidly evolving and can be used in the future as a second-line treatment, IMRT can be applied more actively for glioblastoma for the purpose of preserving immunity.

Reduced TLC and reduced lymphocyte infiltration in pathologic specimens are associated with poor OS [1, 3–5, 7, 26–29]. Several studies have also investigated the association between treatment-related lymphopenia and OS in patients with glioblastoma [5–7], and have shown that radiation-induced reduction of circulating lymphocyte counts and subsequent lymphocyte infiltration of tumors may have a tangible impact on OS outcomes [27, 30]. However, owing to the heterogeneous study population and small number of patients, it was difficult to draw a solid conclusion on the prognostic effect of RT-induced lymphopenia in glioblastoma. In our study, although patients with ASL had poorer OS than did those without ASL, ASL was not a significant factor on multivariate analysis. This inconsistency might be because our study included well-established biomarkers such as *IDH1* mutation, MGMT methylation, and subventricular zone involvement, while previous studies investigated none of these factors [5] or only investigated MGMT methylation [6]. The inclusion of strong prognostic factors [31–33] in our study could have relatively diminished the prognostic significance of ASL. However, when we included grade ≥ 3 lymphopenia at the 3-month time point instead of ASL (grade ≥ 3 lymphopenia at any time point within 3 months) in the multivariate analysis, the results showed that this alternative definition of lymphopenia was a strong independent prognostic factor (Additional file 1: Table S3). This implies that recovery from ASL may be a more important finding than the presence or absence of ASL. However, owing to a lack of consensus and the use of different time points in defining treatment-related lymphopenia across studies, efforts to find the best definition of treatment-related lymphopenia should be continued [30].

The main limitation of this study is that the margins used to define the PTV differed between the IMRT and 3D-CRT groups. Nevertheless, we used the same definition of the GTV and CTV for both groups, and standardized target volume delineation was performed for all patients. The incidence of ASL still showed a significant difference after the PTV was balanced between both groups by using propensity score matching. Because this was a single-center retrospective study, there might have been unrecognized biases that were not completely addressed by multivariate analysis or propensity score matching. Therefore, our findings should be interpreted keeping these limitations in mind.

## Conclusions

This study revealed that although a large PTV can increase the risk of ASL, IMRT can effectively lower the risk of ASL after the initiation of RT plus temozolomide for treating glioblastoma. Our findings add to the growing evidence on the association between RT and treatment-induced lymphopenia in patients with glioblastoma. Particularly, in cases with a large tumor size or surgical cavity, IMRT-based therapeutic strategies should be actively considered to preserve the TLC. Such strategies could potentially improve treatment outcomes in the immuno-oncology era, and would thus need further study.

## Additional file

Additional file 1: **Table S1.** Univariate and multivariate Cox regression analyses for overall survival in the 3D-CRT group. **Table S2.** Univariate and multivariate Cox regression analyses for overall survival in the IMRT group. **Table S3.** Univariate and multivariate Cox regression analyses for overall survival in all patients. **Table S4.** Covariates included in the propensity score matching. **Table S5.** The dosimetric parameters for brain. (DOCX 47 kb)

## Abbreviations

3D-CRT: Three-dimensional conformal radiotherapy
ASL: Acute severe lymphopenia
CI: Confidence interval
CTV: Clinical target volume
GTV: Gross tumor volume
HR: Hazard ratio
IMRT: Intensity-modulated radiotherapy
MRI: Magnetic resonance imaging
OS: Overall survival
PTV: Planning target volume
RT: Radiotherapy
TLC: Total lymphocyte count
