# Supplementary material for: Clinical predictors of radiation-induced lymphopenia in patients receiving chemoradiation for glioblastoma: clinical usefulness of intensity-modulated radiotherapy in the immuno-oncology era
Source: Radiat Oncol. 2019 Mar 27;14:51. doi: 10.1186/s13014-019-1256-6 (PMC6436232; doi:10.1186/s13014-019-1256-6)
Supplement: Supplementary file 1 — Table S1. Univariate and multivariate Cox regression analyses for overall survival in the 3D-CRT group. Table S2. Univariate and multivariate Cox regression analyses for overall survival in the IMRT group. Table S3. Univariate and multivariate Cox regression analyses for overall survival in all patients. Table S4. Covariates included in the propensity score matching. Table S5. The dosimetric parameters for brain. (DOCX 47 kb) [file 13014_2019_1256_MOESM1_ESM.docx]

Additional file 1: Table S1. Univariate and multivariate Cox regression analyses for overall survival in the 3D-CRT group

|  | Univariate HR (95% CI) | *P* |  | Multivariate HR (95% CI) | *P* |
| --- | --- | --- | --- | --- | --- |
| *Patient-related factor* |  |  |  |  |  |
| Age (per 1 year) | 1.02 (1.01–1.03) | 0.008 |  | 1.02 (1–1.04) | 0.011 |
| Sex (Male vs. Female) | 1.07 (0.79–1.45) | 0.668 |  |  |  |
| KPS |  |  |  |  |  |
| (80 vs. ≤70) | 1.01 (0.7–1.48) | 0.938 |  |  |  |
| (≥90 vs. ≤70) | 0.71 (0.48–1.05) | 0.085 |  |  |  |
| Baseline TLC (<1000/μL vs. ≥1000/μL) | 1.29 (0.9–1.84) | 0.159 |  |  |  |
| *Tumor-related factor* |  |  |  |  |  |
| Extent of resection |  |  |  |  |  |
| (Subtotal/Partial vs. Total) | 1.99 (1.45–2.74) | <.001 |  | 2.04 (1.45–2.88) | 0 |
| (Biopsy vs. Total) | 1.33 (0.61–2.87) | 0.475 |  | 1.11 (0.5–2.44) | 0.801 |
| *IDH1* mutation |  |  |  |  |  |
| (Yes vs. No) | 0.36 (0.13–0.99) | 0.048 |  | 0.44 (0.16–1.24) | 0.122 |
| (Unknown vs. No) | 1.2 (0.87–1.64) | 0.270 |  | 1.1 (0.77–1.57) | 0.616 |
| MGMT (Methylated vs. Unmethylated) | 0.54 (0.39–0.74) | <.001 |  | 0.54 (0.39–0.75) | <.001 |
| Subventriclular zone (Involved vs. Uninvolved) | 1.8 (1.31–2.48) | <.001 |  | 1.53 (1.1–2.13) | 0.012 |
| *Treatment-related factor* |  |  |  |  |  |
| The cumulative dose of temozolomide (per 100 mg/m2) | 0.98 (0.97–1) | 0.025 |  | 0.99 (0.98–1) | 0.151 |
| PTV1 volume (per 10 cm^3^) | 1.01 (1–1.01) | 0.189 |  |  |  |
| PTV2 volume (per 10 cm^3^) | 1.02 (1–1.03) | 0.049 |  | 1.01 (0.99–1.02) | 0.405 |
| Total dose (per 1 Gy) | 1.01 (0.96–1.06) | 0.647 |  |  |  |
| No. of fractionation (per 1) | 1 (0.91–1.1) | 0.971 |  |  |  |
| Acute severe lymphopenia (Yes vs. No) | 1.2 (0.89–1.62) | 0.24 |  | 0.94 (0.69–1.29) | 0.713 |

Abbreviations: 3D-CRT, three-dimensional conformal radiotherapy; CI, confidence interval; HR, hazard ratio; IMRT, intensity-modulated radiotherapy; KPS, Karnofsky performance status; PTV, planning target volume; TLC, total lymphocyte count

Additional file 1: Table S2. Univariate and multivariate Cox regression analyses for overall survival in the IMRT group

|  | Univariate HR (95% CI) | *P* |  | Multivariate HR (95% CI) | *P* |
| --- | --- | --- | --- | --- | --- |
| *Patient-related factor* |  |  |  |  |  |
| Age (per 1 year) | 1.01 (0.99–1.02) | 0.263 |  | 1.02 (1–1.03) | 0.07 |
| Sex (Male vs. Female) | 0.99 (0.66–1.5) | 0.979 |  |  |  |
| KPS |  |  |  |  |  |
| (80 vs. ≤70) | 1.18 (0.74–1.89) | 0.482 |  |  |  |
| (≥90 vs. ≤70) | 0.91 (0.56–1.48) | 0.695 |  |  |  |
| Baseline TLC (<1000/μL vs. ≥1000/μL) | 1.2 (0.77–1.87) | 0.413 |  |  |  |
| *Tumor-related factor* |  |  |  |  |  |
| Extent of resection |  |  |  |  |  |
| (Subtotal/Partial vs. Total) | 1.5 (0.96–2.36) | 0.077 |  | 1.43 (0.89–2.29) | 0.137 |
| (Biopsy vs. Total) | 1.91 (1.04–3.49) | 0.036 |  | 1.7 (0.86–3.36) | 0.126 |
| *IDH1* mutation |  |  |  |  |  |
| (Yes vs. No) | 0.24 (0.11–0.55) | 0.001 |  | 0.22 (0.05–0.93) | 0.04 |
| (Unknown vs. No) | 1.14 (0.86–1.51) | 0.360 |  | 0.69 (0.24–2.01) | 0.498 |
| MGMT (Methylated vs. Unmethylated) | 0.46 (0.35–0.6) | <.001 |  | 0.36 (0.22–0.61) | <.001 |
| Subventriclular zone (Involved vs. Uninvolved) | 1.57 (1.23–2.01) | <.001 |  | 1.57 (1.01–2.45) | 0.044 |
| *Treatment-related factor* |  |  |  |  |  |
| The cumulative dose of temozolomide (per 100 mg/m2) | 1 (0.99–1) | 0.260 |  | 1 (0.99–1.01) | 0.502 |
| PTV1 volume (per 10 cm^3^) | 1.01 (1–1.01) | 0.132 |  |  |  |
| PTV2 volume (per 10 cm^3^) | 1.02 (1–1.03) | 0.029 |  | 1.02 (0.99–1.05) | 0.199 |
| Total dose (per 1 Gy) | 0.98 (0.95–1.01) | 0.187 |  |  |  |
| No. of fractionation (per 1) | 0.99 (0.94–1.05) | 0.845 |  |  |  |
| Acute severe lymphopenia (Yes vs. No) | 1.32 (1.03–1.69) | 0.028 |  | 1.21 (0.74–2) | 0.449 |

Abbreviations: 3D-CRT, three-dimensional conformal radiotherapy; CI, confidence interval; HR, hazard ratio; IMRT, intensity-modulated radiotherapy; KPS, Karnofsky performance status; PTV, planning target volume; TLC, total lymphocyte count

Additional file 1: Table S3. Univariate and multivariate Cox regression analyses for overall survival in all patients

|  | Univariate HR (95% CI) | *P* |  | Multivariate HR (95% CI) | *P* |
| --- | --- | --- | --- | --- | --- |
| *Patient-related factor* |  |  |  |  |  |
| Age (per 1 year) | 1.01 (1.00–1.02) | 0.005 |  | 1.02 (1.00–1.03) | 0.017 |
| Sex (Male vs. Female) | 1.02 (0.80–1.30) | 0.889 |  |  |  |
| KPS |  |  |  |  |  |
| (80 vs. ≤70) | 1.06 (0.79–1.42) | 0.697 |  |  |  |
| (≥90 vs. ≤70) | 0.78 (0.58–1.06) | 0.112 |  |  |  |
| Baseline TLC (<1000/μL vs. ≥1000/μL) | 1.25 (0.94–1.64) | 0.119 |  |  |  |
| *Tumor-related factor* |  |  |  |  |  |
| Extent of resection |  |  |  |  |  |
| (Subtotal/Partial vs. Total) | 1.78 (1.38–2.31) | <.001 |  | 1.44 (1.07–1.93) | 0.016 |
| (Biopsy vs. Total) | 1.69 (1.06–2.70) | 0.028 |  | 1.58 (0.91–2.74) | 0.108 |
| *IDH1* mutation |  |  |  |  |  |
| (Yes vs. No) | 0.25 (0.11–0.55) | <.001 |  | 0.26 (0.10–0.63) | 0.003 |
| (Unknown vs. No) | 1.14 (0.86–1.52) | 0.360 |  | 1.06 (0.76–1.47) | 0.729 |
| MGMT (Methylated vs. Unmethylated) | 0.46 (0.35–0.60) | <.001 |  | 0.44 (0.33–0.59) | <.001 |
| Subventriclular zone (Involved vs. Uninvolved) | 1.57 (1.23–2.01) | <.001 |  | 1.54 (1.17–2.04) | 0.002 |
| *Treatment-related factor* |  |  |  |  |  |
| The cumulative dose of temozolomide (per 100 mg/m^2^) | 1.00 (0.99–1.00) | 0.260 |  | 1.00 (0.99–1.01) | 0.947 |
| PTV1 volume (per 10 cm^3^) | 1.01 (1.00–1.01) | 0.132 |  |  |  |
| PTV2 volume (per 10 cm^3^) | 1.02 (1.00–1.03) | 0.029 |  | 1.02 (1.00–1.03) | 0.036 |
| Radiotherapy modality (IMRT vs. 3D-CRT) | 0.91 (0.71–1.17) | 0.483 |  |  |  |
| Total dose (per 1 Gy) | 0.98 (0.95–1.01) | 0.187 |  |  |  |
| No. of fractionation (per 1) | 0.99 (0.94–1.05) | 0.845 |  |  |  |
| TLC <500/μL at 3 months (Yes vs. No) | 2.35 (1.39–3.97) | 0.001 |  | 2.44 (1.42–4.19) | 0.001 |

Abbreviations: 3D-CRT, three-dimensional conformal radiotherapy; CI, confidence interval; HR, hazard ratio; IMRT, intensity-modulated radiotherapy; KPS, Karnofsky performance status; PTV, planning target volume; TLC, total lymphocyte count

Additional file 1: Table S4. Covariates included in the propensity score matching

|  | Before matching | | | |  |  | After matching | | | |  |
| --- | --- | --- | --- | --- | --- | --- | --- | --- | --- | --- | --- |
|  | 3D-CRT (N = 186) | | IMRT (N = 150) | |  |  | 3D-CRT (N = 113) | | IMRT (N = 113) | |  |
|  | N | % | N | % | *P* |  | N | % | N | % | *P* |
| Sex |  |  |  |  |  |  |  |  |  |  |  |
| Female | 96 | 51.6 | 53 | 35.3 | .003 |  | 48 | 42.5 | 44 | 38.9 | 0.678 |
| Male | 90 | 48.4 | 97 | 64.7 |  |  | 65 | 57.5 | 69 | 61.1 |  |
| Extent of resection |  |  |  |  |  |  |  |  |  |  |  |
| Total | 109 | 58.6 | 97 | 64.7 | .009 |  | 71 | 62.8 | 74 | 65.5 | 0.393 |
| Subtotal/Partial | 70 | 37.6 | 38 | 25.3 |  |  | 39 | 34.5 | 32 | 28.3 |  |
| Biopsy | 7 | 3.8 | 15 | 10 |  |  | 3 | 2.7 | 7 | 6.2 |  |
| *IDH1* mutation |  |  |  |  |  |  |  |  |  |  |  |
| No | 117 | 62.9 | 133 | 88.7 | <.001 |  | 99 | 87.6 | 96 | 85 | 0.046 |
| Yes | 6 | 3.2 | 12 | 8 |  |  | 4 | 3.5 | 12 | 10.6 |  |
| Unknown | 63 | 33.9 | 5 | 3.3 |  |  | 10 | 8.8 | 5 | 4.4 |  |
| PTV1 volume, cm^3^, (median, range) | 422 | 74-1080 | 375 | 71-1041 | <.001 |  | 413 | 74–898 | 408 | 93–1041 | 0.136 |
| Baseline TLC |  |  |  |  |  |  |  |  |  |  |  |
| <1000/μL | 45 | 24.2 | 36 | 24.0 | .893 |  | 87 | 77 | 88 | 77.9 | 1 |
| ≥1000/μL | 141 | 75.8 | 114 | 76.0 |  |  | 26 | 23 | 25 | 22.1 |  |
| The cumulative dose of temozolomide, mg/m^2^ (median, range) | 3497 | 856–6267 | 3684 | 722–5599 | 0.117 |  | 3704 | 1524–5818 | 3684 | 674–5599 | 0.822 |

Abbreviations: 3D-CRT, three-dimensional conformal radiotherapy; IMRT, intensity-modulated radiotherapy; PTV, planning target volume; TLC, total lymphocyte count

Additional file 1: Table S5. The dosimetric parameters for brain

|  | Mean±SD | | P |
| --- | --- | --- | --- |
|  | 3D-CRT (n=186) | IMRT (n=150) |  |
| V_0.5 Gy_ (%) | 98.3±6.2 | 97.9±6.9 | 0.766 |
| V_3 Gy_ (%) | 96.6±7.8 | 91.3±13.5 | 0.052 |
| V_5 Gy_ (%) | 95.1±8.4 | 86.8±14.9 | <.001 |
| V_10 Gy_ (%) | 90.7±11.6 | 81.8±18.2 | 0.016 |
| V_25 Gy_ (%) | 70.2±15.3 | 53.6±18.3 | <.001 |

Abbreviations: 3D-CRT, three-dimensional conformal radiotherapy; IMRT, intensity-modulated radiotherapy; SD, standard deviation; Vx = percent brain volume receiving at least x Gy
